# The second wave of COVID‐19 hits Nepal: Burden for Nepal's healthcare system

**DOI:** 10.1002/hsr2.371

**Published:** 2021-10-01

**Authors:** Olivier Uwishema, Kirellos Said Abbas, Tania Torbati, Gana Hamed Abdelrahman, Abayomi Oyeyemi Ajagbe͓, Rabeet Tariq, Hadi Sleiman, Burak Talha Akın, Helen Onyeaka, Shubhika Jain

**Affiliations:** ^1^ Research and Education Oli Health Magazine Organization Kigali Rwanda; ^2^ Department of Research, Education and Projects Clinton Global Initiative University New York New York USA; ^3^ Faculty of Medicine Karadeniz Technical University Trabzon Turkey; ^4^ Faculty of Medicine Alexandria University Alexandria Egypt; ^5^ Western University of Health Sciences College of Osteopathic Medicine of the Pacific Pomona California USA; ^6^ Faculty of Medicine Kafrelsheikh University Kafr El Sheikh Egypt; ^7^ Department of Anatomy, Faculty of Basic Medical Sciences College of Health Sciences, Nile University of Nigeria Abuja Nigeria; ^8^ Department of General Medicine Liaquat National Hospital and Medical College Karachi Pakistan; ^9^ Faculty of Medicine Yeditepe University Istanbul Turkey; ^10^ School of Chemical Engineering University of Birmingham Birmingham UK; ^11^ Department of General Medicine Kasturba Medical College Manipal India

## ETHICS APPROVAL

Not Applicable.

## 1 CORRESPONDENCE

After a relative plateau state of new COVID‐19 cases between December 2020 and April 2021, Nepal is now facing a noticeable increase in the curve of new‐onset COVID‐19 incidents. As of June 28, 2021, Nepal has recorded about 633.679 confirmed COVID‐19 cases since the start of the pandemic. The number of confirmed cases has nearly tripled through the period from May 1 to June 28, 2021—from 238.115 to 633.679. The average weekly new cases have reached 12.623, with 283 deaths per week in June 2021.^1^ This peak overreached to the experiences observed in October and June 2020, not only in numbers but also in disease severity. Of note, the second wave appeared after the emergence of a new COVID‐19 variant in India, intensifying the fear of repeating the Indian scenario in Nepal. Following the entrance of the UK variant of concern in Nepal and given that Nepal shares an open border with India, Ranjit *et.al* strongly advised authorities in Nepal to expand vaccination and to use diverse biotechnological vaccines in order to help control the spread of COVID‐19 variants.^2^ Unfortunately, till June 28, 2021, only 2.62% of Nepal's total population is vaccinated against COVID‐19, posing greater susceptibility and reduced protection against infection for the individuals of Nepal.^1^ In this letter, we will review the COVID‐19 situation in Nepal from various perspectives.

Preliminary to the emergence of COVID‐19 in Nepal, which has a population of 28.087 million people, Nepal has faced an evident scarcity of healthcare workers, including medical doctors, nurses, and paramedics. In particular, healthcare workers have represented only 0.315% of the population since September 2016. Based on governmental data, intensive care beds are available for 1395 people while ventilators are accessible to only 480 individuals, for both COVID‐19 and non‐COVID‐19 infected patients.^3^

The clinical presentations of tuberculosis (TB) and COVID‐19 demonstrate many commonalities, and given the endemicity of TB in Nepal, medical suspension regarding both differential diagnoses has drastically heightened. However, although TB and COVID‐19 present similarly in a clinical setting, minute differences exist as COVID‐19 infection has been shown to occur with a shorter duration of disease than TB. Rapid diagnosis of cases and treatment becomes even more difficult in the light of the COVID‐19 pandemic as seen in other countries.^4^, ^5^, ^6^, ^7^, ^8^, ^9^

India, a prominent neighbor of Nepal, serves as one of the aboriginal countries for TB manifestation, and disease‐positive outcomes from COVID‐19 infection do not eradicate the feasibility and probability of resulting TB. Notably, latent TB infection can lead to severe pneumonia in COVID‐19 patients, increasing the risk of mortality. The central TB Nikshay (Ni = End, Kshay = TB) portal of the Government of India disclosed that diagnosis of new TB cases has decreased by about 78% from 156 000 cases in April 2019 to 34 342 incidents on April 27, 2020 due to challenges imposed by lockdown restrictions in accessing directly observed therapy centers by medical practitioners and sick individuals with TB.^10^ As such, scientific studies have warned against overlooking TB during the COVID‐19 pandemic, especially in the currently aggressive second wave.

The lockdown has also brought about destructive social impacts, including unemployment, increased poverty, amplified inaccessibility to healthcare, and food unavailability leading to undernutrition.^11^ Other major societal concerns include lack of access to maternity health, negative effects on education, reduced surveillance of routine immunization in children, increased domestic abuse, and inadequate obtainable medication.^4^, ^12^ In addition, the prevalence of mental health issues such as anxiety, depression, and stress has escalated significantly.^5^, ^13^ Closure of health services coupled with the lack of awareness, misinformation, and stigma associated with COVID‐19 have instigated limited affordability and fears of COVID‐19 transmission that further contribute to healthcare deficits.^6^, ^14^

Just as the government began easing out of lockdown restrictions and reopening of factories, the increase in COVID‐19 cases in Nepal from India has triggered an alarming concern among the people of Nepal regarding newly implemented lockdown regulations accompanying this second wave. In particular, such guidelines have left harsh effects on the economy. According to scientific literature, Nepal witnessed a negative economic growth of 1.99% last year alone. In addition, the Central Bureau of Statistics has reported that over 1.2 million people moved below the poverty line, and 1.5 million others became unemployed, of which 640 000 were migrant workers, and the rest were jobholders.^15^ Moreover, 10.89% of manufacturers remain closed.^16^

According to WHO, Nepal ranks as third among Southeast Asian countries for new COVID‐19 incidents, with a 137% increase of newly reported cases,^17^ which is extremely worrisome as Nepal is a low‐income country with a fragile healthcare system.^18^ It is recommended that the healthcare system's financing and capacities should afford more flexibility to adapt to emergencies. Furthermore, strengthening and establishing partnerships between private and public sectors and the government are needed. The recruitment process of healthcare workers and other human resources should be organized and thoroughly financed through a long‐term plan that also helps to diminish the spread of COVID‐19 infection, provides attention to other diseases like TB, and prevents further economic injury. Considering the aforementioned information, government, healthcare institutions, physicians, and patients are urged to function with mutual coordination and understanding to enhance the reception of advantageous health services.^3^, ^7^

## FUNDING

We have not received any financial support for this manuscript.

## CONFLICT OF INTEREST

The authors declared no conflicts of interest.

## AUTHOR CONTRIBUTIONS

Conceptualization: Olivier Uwishema

Project administration: Olivier Uwishema

Writing—Original Draft Preparation: Olivier Uwishema, Kirellos Said Abbas, Tania Torbati, Gana Hamed Abdelrahman, Abayomi Oyeyemi Ajagbe͓, Rabeet Tariq, Hadi Sleiman, Burak Talha Akın, Helen Onyeaka, Shubhika Jain

Writing—Review and Editing: Olivier Uwishema, Tania Torbati, Helen Onyeaka

All authors have read and approved the final version of the manuscript.

## Data Availability

Not Applicable.
